# DNA nanotechnology: a future perspective

**DOI:** 10.1186/1556-276X-8-119

**Published:** 2013-03-04

**Authors:** Muniza Zahid, Byeonghoon Kim, Rafaqat Hussain, Rashid Amin, Sung Ha Park

**Affiliations:** 1 Interdisciplinary Research Center in Biomedical Materials (IRCBM), COMSATS Institute of Information Technology, Lahore 54000, Pakistan; 2 Department of Physics & SKKU Advanced Institute of Nanotechnology (SAINT), Sungkyunkwan University, Suwon 440-746, South Korea; 3 Ibnu Sina Institute for Fundamental Science Studies, Universiti Teknologi Malaysia, 81310 UTM Skudai, Johor Darul Ta'zim, Malaysia

**Keywords:** DNA, Nanotechnology, Biomedicine, Nanoelectronics, Nanosensors, DNA computation

## Abstract

In addition to its genetic function, DNA is one of the most distinct and smart self-assembling nanomaterials. DNA nanotechnology exploits the predictable self-assembly of DNA oligonucleotides to design and assemble innovative and highly discrete nanostructures. Highly ordered DNA motifs are capable of providing an ultra-fine framework for the next generation of nanofabrications. The majority of these applications are based upon the complementarity of DNA base pairing: adenine with thymine, and guanine with cytosine. DNA provides an intelligent route for the creation of nanoarchitectures with programmable and predictable patterns. DNA strands twist along one helix for a number of bases before switching to the other helix by passing through a crossover junction. The association of two crossovers keeps the helices parallel and holds them tightly together, allowing the assembly of bigger structures. Because of the DNA molecule's unique and novel characteristics, it can easily be applied in a vast variety of multidisciplinary research areas like biomedicine, computer science, nano/optoelectronics, and bionanotechnology.

## Review

### Introduction

Nucleic acids (e.g., deoxyribonucleic acid (DNA) and ribonucleic acid (RNA)) encode the genomes of all living things on earth. Of these, DNA has become a key biological molecule in the study of genetics, medicine, and biotechnology. It possesses the natural ability to self-assemble and interacts with a wide range of molecules. Besides its importance in genetic studies and its application in various biological fields like biomedicine, cancer research, and genetic engineering, DNA has also become a preferred material for nanotechnologists because of its unique properties of structural stability, programmability of sequences, and predictable self-assembly. Nanobiotechnology is made up of two words: ‘nano’ pertains to the study or development of structures in the 1 to 100-nm size range in at least one dimension, while ‘biotechnology’ refers to technological tools associated with the development of living things or biological molecules. Thus, components of natural biological systems are scrutinized by nanobiotechnologists to engineer innovative nanodevices [1].

**Figure 1:**
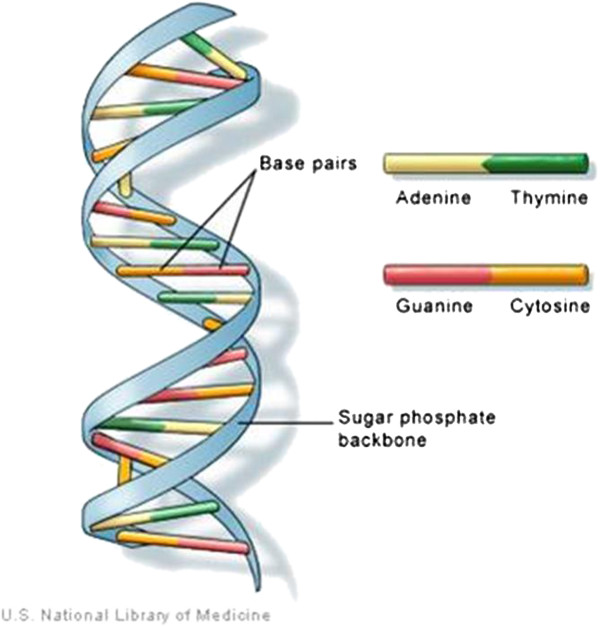
**Basic DNA structure proposed by Watson and Crick.** DNA is made up of two kinds of nitrogenous bases, purines (adenine and guanine) and pyrimidines (thymine and cytosine). Purine bases bind only to their respective pyrimidine bases, i.e., adenine always pairs with thymine, while guanine binds to cytosine [3].

Figure 1 shows the double helical structure of DNA proposed by Watson and Crick in 1953. It primarily consists of nitrogenous base pairs of adenine with thymine (A-T) and guanine with cytosine (G-C), thus offering the advantage of being easily assembled into predictable nanoscale structures by hydrogen bonding. This precision programmability makes DNA an excellent smart material for designing and fabricating nanostructures [2]. Over the last three decades, single and double stranded DNAs have been manipulated to construct branched junction structures in one, two, and even three dimensions with distinct and intricate geometries. The majority of researchers have used a ‘bottom up’ approach of DNA self-assembly to construct dynamic structures.

This has led to the development of several macroscopic structures with nanometer-size features [4-7]. DNA nanotechnology has also been used to produce various kinds of reprogrammable functionalized devices and sensors, some of which will be discussed in this review.

The history of nanoarchitecture is fairly short. In the early 1990s, Seeman and colleagues first described a process by which DNA could be hybridized in more than one way to create self-assembling nanostructures. They created tiles made up of DNA with sticky ends which were allowed to hybridize to form a cube-like structure [8,9]. Yurke et al. experimented with the interesting idea that a single DNA strand can undergo multiple hybridizations through strand displacement cycles using a toehold or hinge made up of the DNA itself. Instead of using proteins and other bio-supportive molecules to build their structures, they demonstrated that DNA strand displacement and hybridization was enough to coax molecular-level changes in the structure of DNA. They achieved this by exploiting two double helical arms of DNA connected by another short DNA sequence acting as a ‘hinge’. This ‘hinge’ repeatedly cycled the two strands into an opened and closed state by consecutive addition of two single-stranded DNA molecules [10]. This method made it possible to form a variety of nanostructures based on differences in sequence, rather than being dependent on the influence of changes in the environment surrounding the DNA (pH, salt, and temperature) [11,12].

DNA-modifying enzymes can also be used to generate and manipulate DNA nanostructures. Although studies in this area have so far been limited, many design tools have been developed for the application of these enzymes to alter DNA in a sequence-specific manner. Most of these enzymes work like small nanofactories and are, hence, highly specific in their actions, based on various biological processes [13].

**Figure 2:**
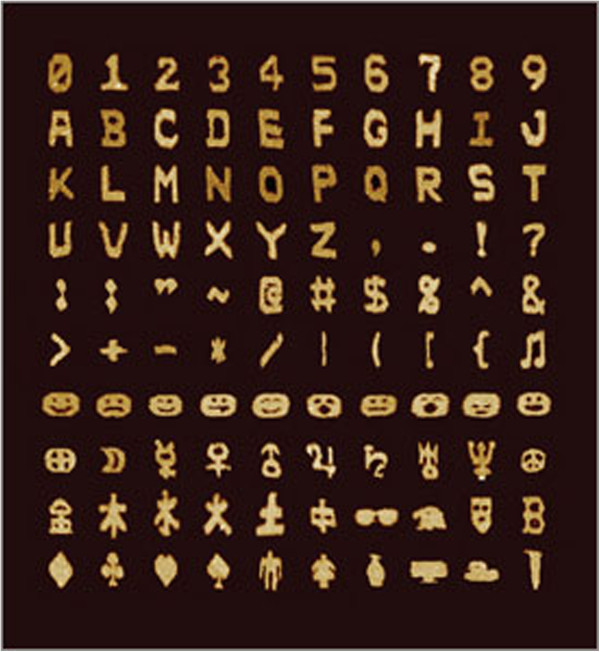
**Complex shapes designed using a DNA molecular canvas.** AFM images of 100 distinct shapes, including the 26 capital letters of the Latin alphabet, 10 Arabic numerals, 23 punctuation marks, other standard keyboard symbols, 10 emoticons, 9 astrological symbols, 6 Chinese characters, and various miscellaneous symbols [26].

The sequence specificity and ease of manipulation of DNA nanoarchitectural structures allow them to carry or organize various biological molecules such as peptides, proteins, and viral capsids [14], as well as complex structures such as carbon nanotubules and other nanoparticles. Such self-assembling DNA nanostructures have increased the activity of enzyme cascades and shifted surface plasmon resonance wavelengths based on their custom-controlled arrangement [15-24]. Nanoconstruction can be used to form structures of various shapes and sizes. Based on the Rothemund model of DNA origami [25], scientists were able to fold long strands of DNA into various interesting two-dimensional shapes depicted in Figure 2[26]. This approach has been very successful so far in producing not only two- but also three-dimensional structures [27-30]. On other occasions, scientists have also employed the use of filamentous viral particles to organize various nanomaterials for short periods of time to form diverse and complex structures which may function as wires, rings, etc. which may have optical, electronic, and biotechnological applications [31,32].

Despite these advances in DNA nanotechnology, it remains in the development phase. Generally, only about 30% of the assembled DNA molecules are similar to the original design [33]. This presents a great challenge for the development of techniques to fabricate modern DNA nanostructures, especially in the DNA computational area. Researchers compare this process with the complicated and eventually successful development of electronics, computers, and automobiles. Besides errors in the ‘designed’ genetic sequences, another shortcoming is that prolonged thermal cycling for up to 24 h is required to produce a useful nanodevice. In case of automobiles, it took over a decade to produce the first functional prototype. Hopefully, the development of potent nanomaterials will not take as long. Here, we review some of the functional challenges and exciting future prospects of developing nanobiotechnology with a special focus on DNA nanotechnology.

### DNA biological applications

**Figure 3:**
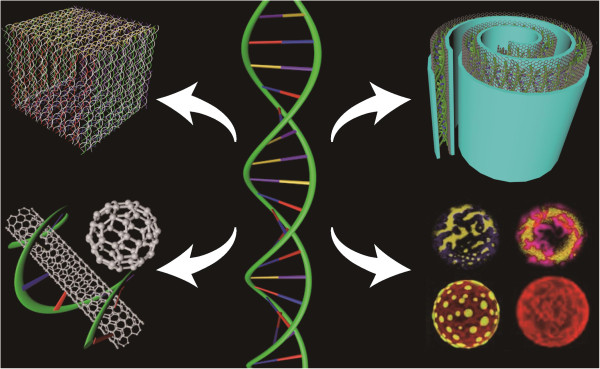
Structural DNA nanotechnology has many applications in modern nanodevice fabrication.

Modern research in nanobiotechnology has offered new hope for its potential application in biomedicine. The physical and chemical properties of nanomaterials such as polymers, semiconductors, and metals present diverse advantages for various *in vivo* applications [34]. Nanobiotechnology provides a new perspective on analytics and therapy in both medicine and pharmacology which has led to the development of a new field called nanomedicine. Various pharmaceutical companies are expanding their research to the application of nanotechnology in vital areas of medicine such as drug delivery and disease therapy [1]. DNA nanotechnology faces several key challenges for its advancement in the future. Nature has developed an intelligent and complex material at the nanoscale through millions of years of evolution. Now, we need time to aggressively pursue new and forward-looking ideas. Along this trajectory of development, advances in structural DNA nanotechnology are expected to allow important progress in the nanotechnology field. Indeed, DNA nanotechnology has already become an interdisciplinary research area, with researchers from physics, chemistry, materials science, computer science, and biology coming together to find solutions for future challenges in nanotechnology. Figure 3 shows the interdisciplinary approaches to DNA nanotechnology and its diverse applications. We believe that more new and exciting directions of research in DNA nanotechnology will emerge in the near future.

### Cancer and nanotechnology

**Figure 4:**
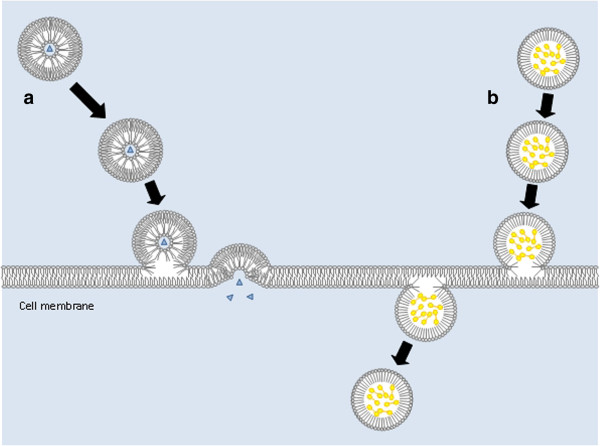
**Drug deliveries by (a) liposomes and (b) emulsions across a cell membrane.** Freely incorporated as well as ligand-bound modes of drug delivery by lipid-based molecules known as liposomes are shown [36].

One of the forefronts of nanomedicine has been the attempt to diagnose, treat, and destroy cancer cells. More than ten million people around the world develop some form of the disease in a single year. Cancer develops when cells begin to function and divide abnormally, not only causing havoc within a particular set of organs but also disrupting the physiology of the entire human body [27,35]. Most cancer therapies require an optimum concentration of chemotherapeutic agents at the tumor site to be able to destroy cancerous cells while diminishing injury to normal cells. Nanotechnology offers several solutions to prevent healthy cell loss as an alternative to chemotherapy. Recent research has focused on the development of technologies such as ligand-targeted delivery of therapeutic drugs and nanocarriers ranging in sizes from 10 to 100 nm. These nanocarriers may be liposomes or albumin-based nanoparticles and were approved for clinical trials by the Food and Drug administration in the United States as recently as 2009 [28,29]. The lipid compositions of liposomes allow them to easily diffuse across cell membranes to deliver therapeutic product to cells (Figure 4).

In addition to the use of liposome-based nanoparticles to carry miniscule amounts of chemotherapeutic agents to affected cancer sites, albumin-bound nanostructures may be used to enhance permeability of the endoplasmic reticulum for breast cancer therapy [29]. Most nanostructures, however, are considered insufficient for effective treatment of cancer cells. This has led to the development of potent ‘nano-systems’, generally possessing four basic qualities: firstly, they can themselves be therapeutic or diagnostic and thus in theory can be designed to carry a hefty therapeutic cargo deliverable to the tumor site. Secondly, more than one targeting ligand can be attached to these nanosystems, providing high affinity and specificity for target cells. Thirdly, these nanosystems have the advantage of being able to house more than one type of therapeutic drug, thereby providing multivalent drug therapy. Finally, most nanosystems that are designed from biological materials such as DNA and RNA are ‘programmed’ to be able to evade most, if not all, drug-resistance mechanisms. Based on these properties, most nanosystems are able to deliver high concentrations of drugs to cancer cells while curtailing damage to surrounding healthy cells [30].

### Drug delivery and biosensors

**Figure 5:**
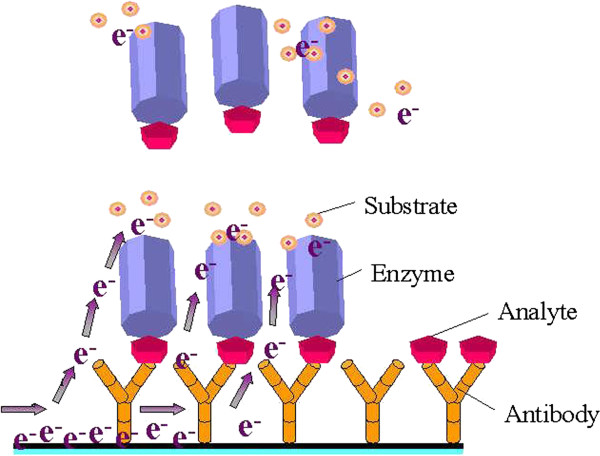
**Schematic illustration of biological sensors used in immunological assays **[39]**.**

Recently, scientists have been able to develop devices that are capable of picking up very specific biological signals and converting them into electrical outputs that can be analyzed for identification. Such devices are known as biosensors [37]. Figure 5 shows a schematic of a biosensor fabrication setup designed to mediate various molecular interactions and to identify minuscule molecular changes with high sensitivity. Unlike macroscopic materials, these biosensors are efficient as they have a high ratio of surface area to volume as well as adjustable electronic, magnetic, optical, and biological properties. Besides having flexible physical structures, these molecules can also be engineered to have diverse chemical compositions, shapes, sizes, and hollow or solid structures. These properties are being incorporated into new generations of drug delivery vehicles, contrast agents, and diagnostic devices [38].

Porous inorganic particles can now be loaded with an assortment of drugs contained in organic nanomicelles that can target very specific cells and tissues in the body. Some of these carbon nanotubules are very potent drug delivery vehicles for cancer treatment [40]. The tubular structure of nanotubules allows for both carrying and protection of drugs from external influences. Therapeutic applications which involve nanomaterials combined with cytotoxic materials such as antineoplastic or chemotherapy agents are a key area of development for science and technology [41].

Research is also being conducted on the use of highly organized DNA lattices to detect biological activity of various molecules. Amin and colleagues have developed a biotinylated DNA thin film-coated fiber optic reflectance biosensor for the detection of streptavidin aerosols. DNA thin films were prepared by dropping DNA samples into a polymer optical fiber which responded quickly to the specific biomolecules in the atmosphere. This approach of coating optical fibers with DNA nanostructures could be very useful in the future for detecting atmospheric bio-aerosols with high sensitivity and specificity [42].

### Dendrimers, enzyme cascades, and contraception

Nucleic acid nanotechnology has many other applications besides medical diagnosis and drug therapy. Synthetic polymers such as dendriworms are made up of dendrimer units of magnetic nanoworms and are being used for intercellular delivery of small interfering RNA (siRNA). These siRNA carriers are assembled from magnetic as well as fluorescent nanoparticles.

**Figure 6:**
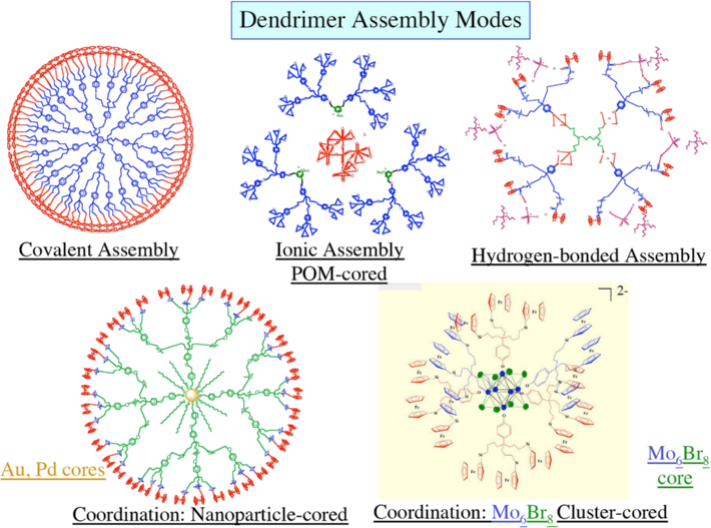
**An assortment of newly assembled structures of dendrimers showing different bonds and metal infusions **[43]**.**

The magnetism of nanoworms allows them to be directed to a particular location, while the fluorescence allows detection. siRNAs are known to be responsible for both activation and silencing of mammalian genes. These siRNAs can be combined with different metals or bound together in diverse ways. Each such assembly may be used to produce contrasting therapeutic effects or to assist drug delivery (Figure 6).

siRNAs have been widely acknowledged as a potent new class of therapeutics, which regulate gene expression through sequence-specific inhibition of mRNA translation. siRNA delivery vehicles such as lipid and polymer nanoparticle-based dendrimers have proven effective in improving the stability, bioavailability, and target specificity of siRNAs following systemic administration *in vivo *[44]. Other important applications have included the activation of enzyme cascades on topologically active scaffolds. This process makes use of DNA self-assembly and uses DNA as a scaffold. Enzymes or cofactor enzymes are attached to this scaffold and then plays an active role in improving the biological efficiency of the system [45]. Bionanotechnology has also been applied in the field of contraception. Where traditional methods have employed over-the-counter drugs and an assortment of widely available contraceptives, bionanotechnology aims to develop drugs that may be effective in targeting the fallopian tubes while anti-implantation drugs can be employed in the uterus to foil pregnancy without influencing other organs. Current studies are centered on manipulating follicle stimulating hormone (FSH) and its inhibitor known as FSH binding inhibitor in mice [46] and monkeys [47].

### DNA computing

DNA computing was first proposed as a means of solving complex problems by Adleman in 1994. He recognized that the incredible storage capacity of DNA could be used to solve complex computational problems. For this, he picked a common mathematical problem normally referred to as the ‘traveling sales man problem’ and was able to solve it using strands of DNA [48]. In 1996, a new technology called the ‘sticker DNA’ model was introduced by Roweis and colleagues. This model applies to random access memory and requires no enzymes or strand extension. This method, thus, has the capability of becoming the universal method for DNA computation. A controlled robotic work station helped not only in implementing the sticker model but also in reducing error rates [49]. Since then, many technologies which make use of DNA to resolve basic mathematical equations and pure computational problems have been developed.

### Mathematical and biological problems

Inspired by Adelman's experiment, researchers have been able to solve a diverse group of mathematical problems using DNA molecules. In 2011, Qian and Winfree were able to calculate square roots using ‘seesaw’ logic gates. The idea behind these gates is that a single stretch of DNA can pair up with various molecules, thus allowing competition for binding sites. Once a molecule is attached, it can be replaced instantly to allow other molecules to fasten themselves to the resident sequence, which itself can be displaced again. This system allows ‘gates’ to be loaded with several input molecules and generates logical output molecules as a result. The various DNA strands can come to represent numbers, of which output can yield the square root result as answers [50].

In another attempt to mimic smart biological computations, the Qian group has developed an artificial neural network. This model employs the use of four neurons. A neuron in its natural environment is susceptible to many incoming inputs, and it ‘reacts’ or ‘fires’ when it reaches a certain threshold. Based on their previous development of logic gates, Qian and his colleagues were able to construct Boolean logical circuits and other circuits which could store memories. The DNA logic circuits were not only able to recall memory using incomplete information but also to determine when conflicting answers were obtained [51]. In other instances, scientists have also used sticker-based DNA to solve the independent set problem [52]. Unlike the earlier sticker DNA system, this model had a random access memory and, thus, required no extension of its strands and enzymes [49].

Inspired by Roweis and Adelman's methods, Taghipour and colleagues [52] set out to unravel the independent set problem through the use of DNA computing. In the beginning, a solution space was created using memory complexes made up of DNA. Then, by the application of a sticker-based parallel algorithm, the independent set problem was solved in polynomial time. Other biological molecules besides DNA have also been used for computation. Faulhammer and colleagues used RNA to solve an assortment of chess problems through DNA computing [53]. Bandyopadhyay and colleagues were able to apply the same reasoning and used 2,3-dichloro-5,6-dicyano-*p*-benzoquinone which is capable of transforming between four different states to mimic natural phenomenon such as diffusion of heat and detection of cancer growth [54].

### Pure computation through DNA

DNA has also been applied for the development of pure computational methods. While many techniques are available to use DNA for computation, the most widely used technique involves the manipulation of mixtures of DNA on a support. A DNA molecule which encodes all possible solutions to a designed problem is synthesized and attached to this supportive surface. Repeated hybridization cycles and action of exonuclease enzymes are used to digest, identify, and eliminate non-solution strands of DNA. Upon completion of this step, several polymerase chain reaction (PCR) reactions are used to amplify remaining molecules, most of which are then hybridized to an array of molecules [55]. Recent progress in DNA computation has been remarkable. Although these advances may be far off to be equivalent of the today's computational capacities of computers, the long-term goal of this research would be DNA computing, overriding everyday computing with great perfection.

### DNA physical applications

The term nanoelectronics refers to the use of nanotechnology for the use and development of electrical components and circuits. Nanoscale electronics have been developed at the molecular level. Such devices are referred to as molecular electronics [56]. Nanoelectronics had been highly dependent on the complementary-symmetry metal-oxide semiconductor (CMOS) technology. CMOS has been vital in analogue circuits such as image sensors, data convertors, and logic-based devices such as digital logic circuits, microcontrollers, and microprocessors [57]. However, CMOS is being replaced as the demand for further miniaturization and processing speeds increase. CMOS circuitry has limitations that can greatly influence the size and shape of computers and other electronics.

**Figure 7:**
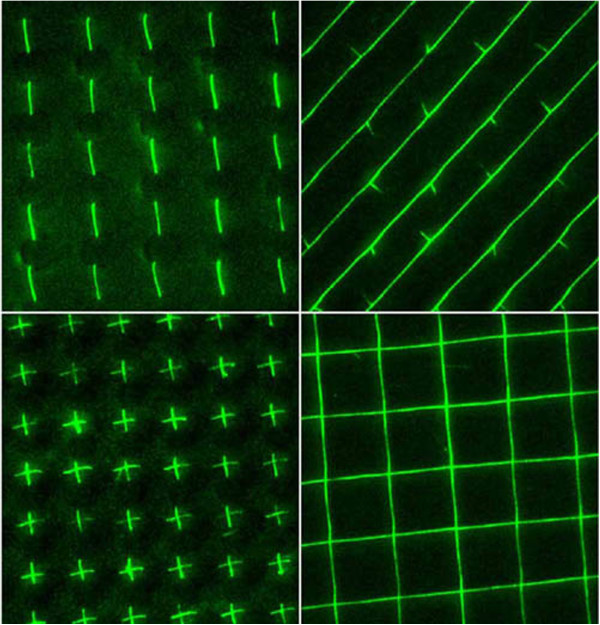
**DNA uncoiling and forming precise patterns, a prelude to biologically based electronics and medical devices **[61]**.**

DNA offers a solution to these problems. Carbon nanotube devices and wires have been developed through self-guided assembly [58]. These materials are capable of forming electronic devices such as nanowires like those shown in Figure 7 and transistors [59,60], thus behaving very similarly to a typical CMOS circuit. The advantage of such devices is that DNA can be accumulated in larger densities and numbers as compared to a typical circuit in a normal electrical system. In addition, DNA is fairly efficient in terms of power consumption and cost [58].

### DNA wires, transistors, capacitors and other devices

**Figure 8:**
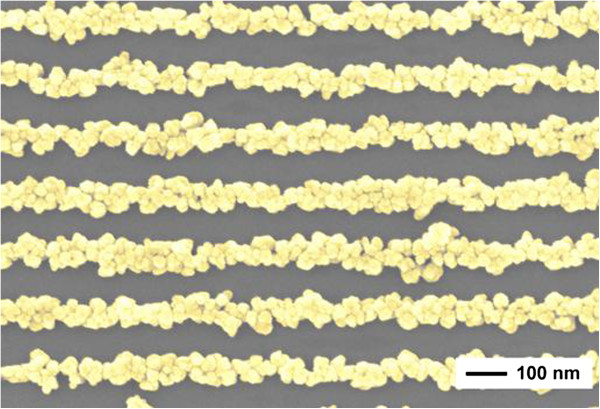
**SEM image of DNA template nanowires.** DNA is used as a template to produce horizontal nanowires. Here, DNA is tagged with a metal such as gold to produce nanowires through self-assembly while being coated onto a niobium oxide surface [64].

DNA self-assembly is essential to form any nanoscale biological device. Prior to the development of nanowires, mostly B-DNA was used. B-DNA has excellent geometrical properties for self-assembly but very limited conductivity at room temperature. Modified DNA (M-DNA) was discovered in 1993 by Lee and colleagues [62]. It was found that the addition of zinc or other divalent metal ions such as cobalt and nickel raised the thermal denaturing temperature at a high pH of 9. The addition of zinc at high pH suggested that a new conformation was formed. This structure is a good conductor compared to B-DNA molecules as the M-DNA duplex is a chain of metals surrounded by an organic sheet and, hence, capable of electron transport. Thus, M-DNA can be considered as a nanowire [63]. Figure 8 is a representation of a scanning electron microscopic image of a nanowire made up entirely of DNA [64].

**Figure 9:**
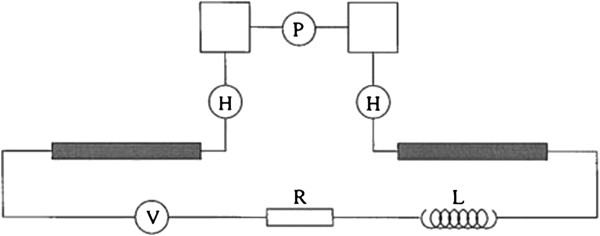
**A qubit made of one short DNA strand attached to two long strands by two H-bonds.** The long strands are metal-coated and connected to an external voltage source, *V*, via resistance, *R*, and inductance, *L*[67].

Fink and Schönenberger extended this rationale to a single DNA rope which consisted of a few molecules. They measured the current conducted through the DNA with a potential applied across the DNA under high-vacuum conditions at room temperature as shown in Figure 9. The charge transport mechanism was, thus, determined to be electronic in nature [65]. In another experiment by Porath and colleagues, the voltage applied across the DNA was about 4 V between two platinum nanoelectrodes, and the resulting current did not surpass 1 pA below the threshold voltage of a few volts. This showed that the system behaved as an insulator at low bias. However, beyond the threshold, the current sharply increased indicating that DNA could transport charge carriers [66].

Various spectroscopic methods were also used to investigate DNA conductivity. The movement of electrons was detected at the level of single molecules by fluorescence decay. Varying fluorescence levels indicated how electrons may have been transferred along the DNA chains [68,69]. Contact methods can be used to measure conductivity directly. Molecules are laid directly on top of gold electrodes, and current flowing across these circuits is plotted on a graph to ascertain levels of conductivity. However, with this method, it is often difficult to determine whether DNA molecules are in direct physical contact with the electrodes. It is thought that weak physical contact between the DNA and electrode produces an insulating effect and, thus, accounts for varying resistance across the circuit. An expansion in experimental methodology to measure conductivity by a contactless approach will improve understanding of this process [70].

**Figure 10:**
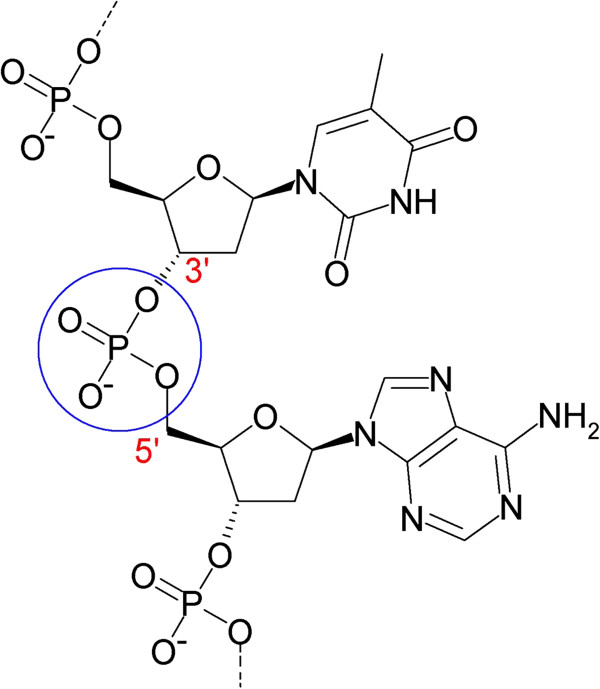
**Representation of the phosphate bonds in a DNA transistor.** The phosphate group forms a P-bond between two sugars, which acts as a tunneling junction between the sugars [73,74].

Recently, researchers have been able to develop electrical units besides wires, such as DNA-based transistors [67,71]. In 1999, Ben-Jacob and colleagues [67] started to build the world's first DNA-based transistor. Figure 9 is a unit representation of the DNA transistor [4]. To do this, they began by joining two DNA strands. These were assigned as a main strand and a gate strand. The end base of the gate strand was connected to the middle of the main strand. Both strands were metal-coated (as that is important for conductivity) except for the middle region of the main strand. This middle region was connected to the gate strand as well as to two adjacent phosphate bonds. The subsequent connecting hydrogen bonds were also left uncoated. It is important to mention that these strands were artificially synthesized so that both coated and non-coated regions were made up of very specific but unique sequences of nucleotide bases [67]. The ends of the DNA strands, which were coated with metal ions were connected to a voltage source, *V*, as well as to another voltage source, *V*_G_, which could act as the gate voltage. This DNA device, thus, acted as a single electron transistor [72]. Figure 10 below shows a pictorial representation of this process [73,74].

This model is essentially a grain connected by two tunnel junctions to a voltage source. The DNA molecule is not very conductive; however, it does possess a large energy gap which makes single electron transfer possible. In order for this circuit to operate as a transistor, the voltage supplied to the circuit is varied around threshold levels. This voltage can be varied if the tunneling rates of electrons between the two junctions are different or if there is a gap in the density of the energy states of the grain. The natural energy gap of the DNA can be enhanced using a longer strand of DNA having more than one grain. Longer chains of DNA tend to have more non-linear effects. As a result, more charges are formed. A large uncoated DNA molecule is, thus, used as compared to one that is entirely coated with a metal sheath. The tunneling rates of electrons, however, are about the same as the two phosphate bonds are identical. To counter this effect, a chemical group may be attached to one of the phosphate bonds, thus altering its properties and making electron transport and transistor behavior possible [67].

Some studies have reported the formation of three-dimensional structures such as switches [75] and motors [11]; devices such as DNA-based capacitors are also being contemplated. Biological polymer-based DNA hybrids have intriguing electrical characteristics such as a high dielectric constant, dielectric breakdown behavior, and good resistivity. These are encouraging signs for the development of DNA-based capacitors [76]. In another DNA-bioploymer-based study, Nakamura and colleagues developed a light-emitting diode based on a DNA/polyaniline/Ru(bpy)_3_^2+^ and *tris*(8-hydroxyquinolinato)aluminum complex. The voltage across the hybrid circuit was increased from 5 to 14, 16, and finally 18 V. The light emitted varied in color, ranging from green, yellow, orange, and finally to red. This was the result of electron transfer in the DNA hybrid molecule with increasing voltage [77]. Other important DNA-based nanoscale devices that have recently been developed include highly conductive nanowires [78], quantum dots with carbon nanotubules [79], and even radically advanced devices which detect single-nucleotide polymorphism and conduct nucleotide sequence mutation analysis [80]. With added progress in this field, it could be possible to use DNA-based electronics for both DNA-based diagnostics and sophisticated nanoscale electrical devices.

### DNA optoelectronics

**Figure 11:**
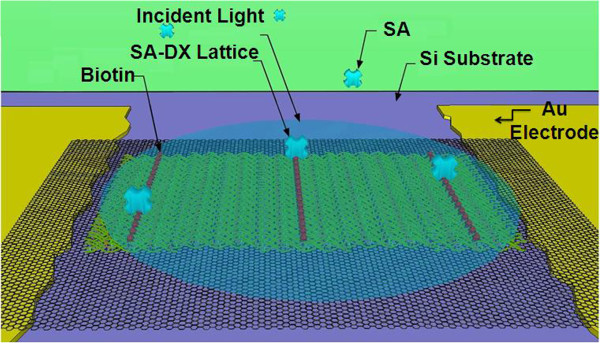
**Schematic of the biotinylated DNA lattice structure layered onto a graphene sheet connecting two gold electrodes, with streptavidin binding to the biotin protein **[85]**.**

With recent advances in the field of biological electronics, there is great interest in developing problem-solving novel nanodevices for detection [81,82], diagnosis [83], and discovery [84]. These devices may be used for a variety of purposes. Nano-optoelectronics is the field of applying light to achieve or modify various biological functions at the DNA or protein level. Kulkarni and colleagues recently attempted to do just that by demonstrating the ability of photons to induce conductivity in two-dimensional DNA nanostructures with and without the help of graphene (Figure 11) [85]. They proved that the conductivity of DNA lattices lined with streptavidin protein could be further improved by the addition of graphene sheet [85]. This optical pulse response of the DNA to graphene is very encouraging and may be exploited in the construction of biological sensors for immunological assays, DNA forensics, and toxin detection.

In another study, Kim and colleagues attempted to construct a biosensor based on graphene and polydimethylsiloxane (PDMS) [86]. An evanescent field shift occurred in the presence of chemical or biological structures which were very sensitive in the refractive index. They were able to monitor the target analyte by attaching the selective receptor molecules to the surface of the PDMS optical waveguide resulting in a shift of the optical intensity distribution. Hence, they monitored the electrical characteristics of graphene in the dark and under PDMS wave-guided illumination. Changes in the resulting photocurrent through the graphene film showed that the fabricated graphene-coupled PDMS optical waveguide sensor was sensitive to visible light for biomolecular detection [86]. This finding can be used for the development of optical biosensor for the detection of various biological molecules in future biological assays.

### Correction of sequence mismatch

The rise of DNA-based nanobiotechnology has led to an increase in demand for synthetic DNA. DNA can be synthesized from nucleotides into small molecules such as ssDNA up to entire viral genomes. In spite of these accomplishments, the time and cost of synthesizing such molecules have somewhat limited the use of DNA as a current research tool. Another significant drawback in this technology has been the significant error rate of synthetic DNA sequences [87]. The reduction and correction of errors are, thus, essential for the synthesis of long DNA molecules. The correction of these errors is, however, very time-consuming and expensive. There are several approaches to develop error-free sequences in synthesized populations of DNA.

These methods may include, but are not limited to, physical separation which may apply the use of metals to chelate partially denatured purine bases and allow elimination of errors [88] or PCR-based approaches such as hairpin PCR, which completely separates genuine mutations from polymerase mis-incorporations. Hairpin PCR operates by converting a DNA sequence to a hairpin following ligation of oligonucleotide caps to DNA ends. Conditions are such to allow a DNA hairpin to be efficiently PCR‐amplified so that during DNA synthesis, the polymerase copies both DNA strands in a single pass. Consequently, when a mis-incorporation occurs, it forms a mismatch following DNA amplification and is distinguished from genuine mutations that remain fully matched [89].

**Figure 12:**
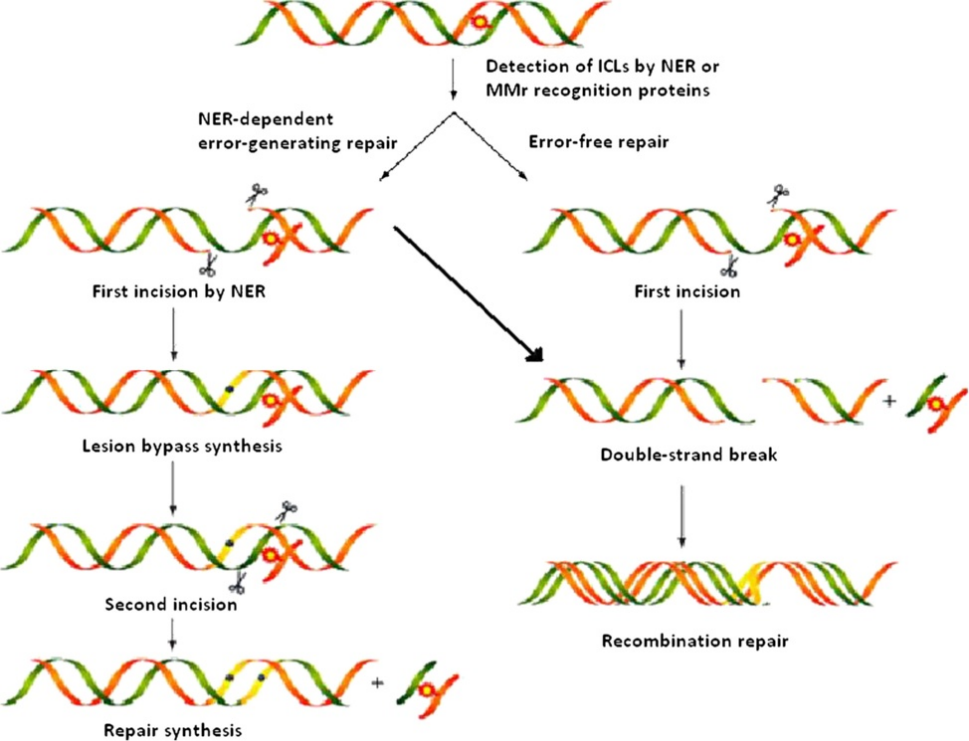
**Mismatch repair mechanism of synthetic DNA to produce error-free DNA.** Representation of an inter-strand repair mechanism which involves mismatch repair, excision repair, and homologous recombination [91].

Sequential errors have also been removed using ‘selective destruction’ methods. Smith and Modrich employed the use of MutH, MutL and MutS mismatch repair proteins under double-strand cleavage conditions, followed by isolation of uncleaved product by size selection. This technique has allowed them to reduce the number of mutations in PCR products and reduce errors [90]. In another instance, Young and colleagues combined dual asymmetrical PCR and overlap extension PCR, which enables any DNA sequence to be synthesized error free. For PCR-based purification methods, gel electrophoresis and cloning is performed. However, the existing approaches are not well suited for error removal in long synthetic DNA sequences where virtually all members in the population contain multiple errors [91] as shown in Figure 12.

New approaches in the production of error-free DNA exploit the use of self-assembly and natural error correction proteins. Among these proteins, celery I nuclease enzyme (CEL I; Surveyor, Transgenomic, Inc., Omaha, USA) endonuclease has been very useful [92]. Hughes and colleagues [92] found CEL I to be a reasonably effective at reducing synthetic DNA errors up to six times. The enzyme is added to previously amplified PCR product, and this mixture is subjected to a second round of thermal cycling at the end of which it is put through gel electrophoresis, quantified, and cloned. CEL I is a naturally occurring enzyme that cleaves mismatched DNA sequences [93-95]. It is, thus, most effective at removing common insertions and deletions that may occur during DNA synthesis [96].

Another tactic in dealing with error-prone DNA synthesis is changing the way we synthesize premeditated DNA. Usually, the formation of synthetic DNA requires the use of PCR-based technologies, but microarrays are now also used to synthesize DNA [97]. In this case, DNA synthesis typically relies on spatial confinement of reactions to certain regions on a silica chip since this technology employs the addition of picoliters of reagents to the silica chip. Error rates can be reduced by controlling the locations on the chip where the reagents eventually end up. Another possibility could be directing reacting reagents through the use of photochemistry. In this way, light can be used to block or restrict reactions at potential error sites. Directing redox reactions only at desirable sites in the forming DNA is another approach. All these strategies can help reduce error rates from 1 in 200 bases to 1 in 600 bases [98].

## Conclusion

DNA is one for the most useful engineering materials available in nanotechnology. It has the potential for self-assembly and formation of programmable nanostructures, and it can also provide a platform for mechanical, chemical, and physical devices. While the formation of many complex nanoscale mechanisms has been perfected by nature over the course of millennia, scientists and engineers need to aggressively pursue the development of future technologies that can help expand the use of DNA in medicine, computation, material sciences, and physics. It is imperative that nanotechnology is improved to meet the need for better detectors in the fields of biological and chemical detection and for higher sensitivity. In terms of DNA-based nanostructures, there is an urgent need to develop sophisticated architectures for diverse applications. Currently, much progress is being made in modelling DNA into various shapes through DNA origami, but the next step is to develop intelligent and refined structures that have viable physical, chemical, and biological applications. Despite the fact that DNA computation may be in its infancy with limited forays into electronics and mathematics, future development of novel ways in which DNA would be utilized to have a much more comprehensive role in biological computation and data storage is envisaged. We are hopeful that the use of DNA molecules will eventually exceed expectations far beyond the scope of this review.

## Abbreviations

A: Adenine; C: Cytosine; CEL I: Celery I nuclease enzyme; CMOS: Complementary metal-oxide semiconductor; DNA: Deoxyribonucleic acid; FSH: Follicle-stimulating hormone; G: Guanine; PCR: Polymerase chain reaction; PDMS: Polydimethylsiloxane; siRNA: Small interfering ribonucleic acid; T: Thymine.

## Competing interests

The authors declare that they have no competing interests.

## Authors’ contributions

MZ, RA, and SHP defined the theoretical framework of the study. MZ and RA gathered the research data. RA, SHP, BK, and RH analyzed these data findings and contributed to the conclusions. All authors read and approved the final manuscript.

## Authors’ information

SHP is working as an assistant professor in the Department of Physics and SKKU Advanced Institute of Nanotechnology (SAINT) at the Sungkyunkwan University, Suwon, Korea. His research interests span experimental nanobio sciences including but not limited to physical and biological circuit design and device fabrication using nanoscale materials; design, fabrication, and testing of micro/nanomechanical devices; electrical and mechanical characterization of circuits, sensors and devices; and biophysics, especially in DNA bottom-up self-assembly and its applications. RA is working as an assistant professor in the Interdisciplinary Research Center in Biomedical Materials (IRCBM) at COMSATS Institute of Information Technology, Lahore, Pakistan. His research interests are in the field of artificially designed DNA nanostructures and their applications in different fields, especially in biosensor applications, nanodevices designing and fabrication, and tissue engineering, especially in assisting burn patients.
